# Anonymisation of geographical distance matrices via Lipschitz embedding

**DOI:** 10.1186/s12942-015-0031-7

**Published:** 2016-01-07

**Authors:** Martin Kroll, Rainer Schnell

**Affiliations:** Research Methodology Group, University of Duisburg-Essen, Lotharstraße 65, 47057 Duisburg, Germany; City University London, Northampton Square, EC1V 0HB London, UK

**Keywords:** Masking, Geographical data, Distance matrix, Privacy, Disclosure risk, Confidentiality

## Abstract

**Background:**

Anonymisation of spatially referenced data has received increasing attention in recent years. Whereas the research focus has been on the anonymisation of point locations, the disclosure risk arising from the publishing of inter-point distances and corresponding anonymisation methods have not been studied systematically.

**Methods:**

We propose a new anonymisation method for the release of geographical distances between records of a microdata file—for example patients in a medical database. We discuss a data release scheme in which microdata without coordinates and an additional distance matrix between the corresponding rows of the microdata set are released. In contrast to most other approaches this method preserves small distances better than larger distances. The distances are modified by a variant of Lipschitz embedding.

**Results:**

The effects of the embedding parameters on the risk of data disclosure are evaluated by linkage experiments using simulated data. The results indicate small disclosure risks for appropriate embedding parameters.

**Conclusion:**

The proposed method is useful if published distance information might be misused for the re-identification of records. The method can be used for publishing scientific-use-files and as an additional tool for record-linkage studies.

**Electronic supplementary material:**

The online version of this article (doi:10.1186/s12942-015-0031-7) contains supplementary material, which is available to authorized users.

## Background

The amount of microdata gathered by governmental, research, and other institutions has increased considerably within the last decades. Due to laws such as the American Freedom of Information Act and the principles of good scientific practice, more and more of these datasets are available for secondary analyses. In many research fields such as medicine or social research, microdata files contain information about individuals. But the units of observation in a microdata file might also be hospitals and other health care providers, schools or households. Today, many data sets available for secondary analyses already contain location information for the units of observation. Given such data, techniques from spatial statistics can be used to approach research problems such as disease clustering and their causes [1].

### Anonymity of spatial data

In principle, the anonymity of research data on individuals and organisations should be guaranteed. Therefore, the release of microdata is strongly regulated in most countries. In general, it is required that the re-identification risk of anonymised records should be very small. Of course, the technical details to comply with national legal requirements vary between countries and may be open to interpretation, as for example in the case of the American HIPAA rules [2].

The re-identification problem of anonymised records is discussed in the technical literature on *statistical disclosure control*. There, a distinction between *attribute* and *identity disclosure* is made [3]. As in the majority of published papers, we will focus on identity disclosure: We consider the risk of re-identification of at least some of the sampling units whose data are published.

Re-identification is much easier if spatial information for the observational units is available in the published data. El Emam and Arbuckle point out that *location is often one of the critical pieces of information for a successful re-identification attack* (see [4], p. 127). This issue has been addressed in various research papers (see “Previous work” section below). Most of these contributions attempt to preserve the spatial distribution of units within the corresponding geographical areas and the underlying areas themselves. Although attacking a file containing geo-masked coordinates is more difficult than attacking an unmasked file, all masking methods for coordinates permit the computation of a (approximate) distance matrix. This distance matrix can also be used for an attack, for example by the attack described in the “A graph theoretic linkage attack” section. Therefore, encrypting coordinates as, for example, by the method described in [5], may not be sufficient. It should be mentioned, that a distance based attack can also be successful (for small files) if no additional information for a person (for example, age or sex) is available: Only a file with identifiers and a corresponding distance matrix are needed.

We focus on the preservation of distances only, for example the geographic distances between the occurrences of a given disease in a population. It is intuitively appealing that dispensing with the underlying geographical area for anonymisation might permit the release of more accurate distance information.

Since the distance matrix can be computed from the geographical coordinates, it is evident that releasing even perturbed location data offers a potential attacker more information than the release of an approximate distance matrix. Given any extra information (e.g., perturbed coordinates), our attack can still be performed or even improved. Although the release of distance matrices instead of coordinates makes re-identification attacks more difficult, releasing the exact distance matrix *D* might give a potential attacker sufficient information for an attack. Therefore, releasing a modified version $\widetilde{D}$ of the original distance matrix *D* will hopefully make re-identification unreasonably difficult. The proposal of a new modification method for the release of distance matrices accompanied by microdata and the empirical study of its privacy are the topics of this paper.

### Previous work

Anonymisation of spatial data has been addressed in many research contexts (for example, see [6]). Comprehensive reviews of the available methods have been given by Armstrong et al. [7] and O’Keefe [8].

Following [9], the methods sketched in [7] can be sorted into three categories: (1) methods that aggregate spatial points, (2) methods that modify coordinates, and (3) methods that release contextual data only. Examples of the first category include point and areal aggregation. Translation, rotation, scaling and random perturbation belong to the second group, whereas the release of the distances to nearest neighbours provides an example of the third category.

Due to its simplicity, aggregation is the most popular method for releasing administrative or health data containing spatial references. Using areal aggregation, a sufficient level of confidentiality can often be achieved. For example, the US HIPAA rules 45 CFR 164.514 demand aggregation of ZIP codes. However, the protective effect of aggregation is coupled with a massive loss of precision in the calculation of distances, especially for entities in close proximity. Therefore, the problem of choosing suitable aggregation units has received a lot of attention [10, 11].^1^

A special case of random perturbation is proposed in [12]. In that paper the authors suggest moving each point into the area of an annulus centered at this point. Both the inner and the outer radius of the annulus are determined as dependent on the relative population density such that the chosen anonymity requirement (*k*-anonymity in this case) is satisfied. The authors show that their approach outperforms aggregation concerning cluster detection under the privacy requirement of *k*-anonymity. Moreover, they show that their approach suffers from a minimal loss in cluster detection performance compared with random perturbation but yields a considerably higher degree of privacy protection.

Another innovative strategy for the anonymisation of spatial point data is due to Wieland et al. [13] who developed a method based on linear programming which moves each point in the data set as little as possible under a given quantitative risk of re-identification. A modification of this technique for small data sets was suggested in [14].

However, this approach also intends to preserve the spatial distribution of the sampling units, whereas our focus is on releasing spatial information only through the distance matrix (in addition to microdata without coordinates).

In the literature, the preservation of distances has been mentioned only in passing. A notable exception is the paper by Kerschbaum [15] which focuses exclusively on distance preservation. In that paper, a regular grid of reference points is generated and a hash value is assigned to each of these grid points. For a given point location hash values of adjacent grid points and further numerical measurements (for example, distances to adjacent grid points) are stored. From this information the distance between two points can be exactly recovered if their distance is smaller than a threshold *d* which depends on the acuteness of the grid. If the distance is greater than 2*d* no distance can be computed.

Our method does not use a regular grid but makes use of reference sets whose elements are randomly sampled points. Moreover, even small distances are preserved only approximately by our method rather than exactly which contributes to the resilience of our method against attacks.

### Examples of statistical procedures compatible with the new anonymisation method

If the intended analysis requires the computation of distances, the necessary information is given by the distance matrix *D* containing the pairwise geographic distances between the units of observation. Using this kind of information is sufficient for statistical analyses of many research problems. For example, methods for the detection of spatial clusters of infectious diseases are important in epidemiology. In this context, the article [16] introduces a test whose test statistic does only depend on the interpoint distances and thus can be calculated from the distance matrix only.

In general, the new method described in this article is intended for statistical analyses of inter-record distance matrices in combination with additional attributes. Such data are widely available in infectious disease modeling, environmental epidemiology and socio-geographics. We discuss a data release scheme in which the microdata without coordinates and an additional distance matrix between the rows of the microdata set are released. We assume that the one-to-one correspondence between the rows of the microdata set and the rows/columns of the distance matrix is known. Examples of applicable methods are agglomerative clustering algorithms (see [17], ch. 4) and nearest neighbour imputation (see [18], p. 52). Another example is geographically weighted regression (GWR) since the computation of GWR requires only microdata and a weight matrix. Since the weight matrix can be computed from the distance matrix (see [19], p. 123, eq. (5.35)), our method can be used along with GWR as well. Furthermore, methods based on truncated distance matrices such as PCNM [20] can be used with our anonymisation procedure. The same is true for indices of spatial autocorrelation such as Moran’s I or Geary’s C. Concerning spatial information, the computation of such quantities is based on a spatial weight matrix which can be computed from the distance matrix (see [21], ch. 7.4). If the definition of the weight matrix is based on contiguity, it can be shown empirically that spatial autocorrelation indices will be approximated well.

## Methods

We introduce a new technique for generating an anonymised version $\widetilde{D}$ of a spatial distance matrix *D* to be released in addition to corresponding microdata. After the description of the technique in the “Contractive anonymisation of spatial point data” section, the accuracy of the resulting distance approximations is discussed analytically and using examples in the “Accuracy of the proposed method” section.

### Contractive anonymisation of spatial point data

We assume the following situation: A data holder *Alice* is willing to release microdata including geocodes that permit useful distance approximations between the observational units. Thus the published data should be available to any researcher (for example, *Bob*) who wants to perform analyses based on this data. Hence, the data must be sufficiently anonymised by Alice such that re-identification of the observational units by a malicious adversary *Eve* is only a remote risk.

We assume that Alice has already created a sufficiently anonymised version *T* free from any spatial reference of the original database $T_0$ by using the variety of prevailing methods for this purpose. Furthermore, we assume that for each record $t_i$ of *T* a geographic point datum $p_i$ is known to Alice. For instance, the original database $T_0$ could have contained the household addresses of patients and their corresponding geographic coordinates.

Let $N \in \mathbb N$ denote the number of rows in *T*. The exact distances between the entities in *T* are stored in an $N \times N$-matrix $D=(d_{ij})$ where $d_{ij}$ is the distance between the *i*-th and the *j*-th record in *T*. The output of our method consists of an $N \times N$ distance matrix $\widetilde{D}$, which is an approximate version of *D* more suitable for being released in addition to *T*.

**Fig. 1:**
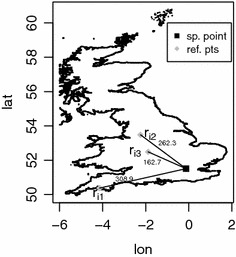
Illustration of step 3. The size parameter *k* is chosen equal to 3 and the elements of the reference sets are sampled at random from the administrative area of the United Kingdom. The coordinate $f_i(p)$ of the point *p* (*black square*) with respect to the random reference set $R_i=\{r_{i1},r_{i2},r_{i3}\}$ is given by the minimum distance from *p* to a point of this reference set. We have $d(p,r_{i1})=308.9$, $d(p,r_{i2})=262.3$ and $d(p,r_{i3})=162.7$, thus $f_i(p)=\min \{308.9, 262.3, 162.7\}=162.7$. All distances are measured in kilometers

Our algorithm depends on two embedding parameters: $d \in \mathbb N$ (the *dimension parameter*) and $k \in \mathbb N$ (the *size parameter*). The effect of these parameters on the accuracy and the provided anonymity will be studied below. The algorithm consists of the following steps:

1. Choose the embedding parameters *d* and *k*.
2. Create *d* random reference sets $R_1,\ldots ,R_d$ of size *k*, i.e. $R_i=\{r_{i1},\ldots ,r_{ik}\}$ for $i=1,\ldots ,d$. The elements $r_{ij}$ of the reference sets shall be drawn independently and uniformly from a geographical area $\mathcal A$. For the rest of this paper we assume that $\mathcal A$ coincides with the geographical area from which the spatial point data considered are taken, although other choices are possible.
3. In this intermediate step each point location *p* is mapped to $\mathbb R^d$ via
  $$\begin{aligned} p \mapsto f(p):=(f_1(p),\ldots ,f_d(p)) \in \mathbb R^d \end{aligned}$$
  where the coordinate functions $f_i$ are defined by
  1 $$\begin{aligned} f_i(p):=\min \limits _{j = 1,\ldots ,k} d(p,r_{ij}) \end{aligned}$$
  and *d*(*p*, *r*) is the distance between $p_i$ and *r*. This step is illustrated in Fig. 1.
4. The approximate distance $\widetilde{d}(p,q)$ between two point locations *p* and *q* is computed as the $\ell _\infty$-distance between the embedded points *f*(*p*) and *f*(*q*) in $\mathbb R^d$, i.e.
  2 $$\begin{aligned} \widetilde{d}(p,q) := \Vert f(p)-f(q)\Vert _\infty =\max \limits _{i=1,\ldots ,d} \vert f_i(p)-f_i(q) \vert . \end{aligned}$$
5. The output is the pair $(T,\widetilde{D})$ where $\widetilde{D}=(\widetilde{d}_{ij})$ and the $\widetilde{d}_{ij}$ are defined through $\widetilde{d}_{ij}:=\widetilde{d}(p_i,p_j)$.

An exemplary implementation of the above algorithm in the programming language R [22] is provided in Additional file 1: Appendix of this article.

*Remark* The embedding into $\mathbb R^d$ applied in step 3 above is a variant of a technique which is commonly denoted as *Lipschitz embedding* [23]. Lipschitz embeddings have been used in other scientific areas, for example to reduce the number of dimensions (for example in biochemistry [24]) or to provide a vector-based representation of non-vectorial data [25]. Note that in our proposal, Lipschitz embedding is not used to obtain a simplified representation of the given data, but as a technique for distance modification. In particular, the Lipschitz coordinates $f_i(p)$ generated in step 3 of the algorithm are not released at all and therefore cannot be used by an attacker to re-identify entities from the pair $(T, \widetilde{D})$.

In the field of data privacy, the use of Lipschitz embedding has been suggested for privacy-preserving string comparisons [26]. Furthermore, other embedding techniques such as the Johnson-Lindenstrauss embedding have been studied in other settings [27] where data privacy is essential.

### Accuracy of the proposed method

In general, accuracy of distance computations is linked with the provided degree of anonymity and vice versa. More accurate released distances will give more information to the adversary. Therefore, the attacker will study the amount of error in distance calculations caused by the embedding technique.

Before considering the effect of the embedding parameters *d* and *k*, we state a fundamental property of Lipschitz embeddings.

#### **Proposition 1**

(Contractivity of Lipschitz embedding) *We have*$\widetilde{d}(p,q) \le d(p,q)$*for all p*, *q, so that the approximate distance*$\widetilde{d}(p,q)$*never exceeds the original distanced*(*p*, *q*).

For a proof of Proposition 1 we refer the reader to [28]. The contractivity of Lipschitz embedding is a well-known fact and of importance because it is a desirable property for data analysis techniques such as clustering [24]. The results of cluster analysis are only slightly affected by the embedding, because the metric space formed by the relevant points is compressed, not distorted. Most other geo-masking methods such as aggregation, random pertubation or donut-transformation do not have this contractivity property.

The statement of Proposition 1 is rather imprecise because it does not describe to what extent distances between pairs of points are transformed in dependence on the embedding parameters *d* and *k*. Hence, it is important for the adversary Eve to study the effect of *d* and *k* on the accuracy of the transformed distances.

The dependence of the accuracy of transformed distances on the dimension parameter *d* is obvious: As *d* increases, the maximum in (2) is taken over more independent realisations $\vert f_i(p)-f_i(q) \vert$ of the same random variable. Since $\widetilde{d}(p,q)$ is bounded from above by *d*(*p*, *q*) due to Proposition 1, a first conjecture is obvious:

1. The approximation of distances is likely to become better if *d* increases.

Let us now consider the dependence on the parameter *k*. We denote a point for which the minimum over all $r_{ij} \in R_i$ in (1) is attained as an *anchor point*. For $k=1$ the anchor point must be the same (namely $r_{i1}$) for all *p* and all reference sets $R_i$. Albeit the coincidence of anchor points for different point locations *p* and *q* does not guarantee that their distance is accurately approximated (the approximate distance can even be 0 in this case), it is easy to see that it makes accurate approximation more likely. For $k \gg 1$, it is likely that the corresponding anchor points differ for many reference sets if *p* and *q* are far away from each other. Under this condition, original distances will be underestimated.

This reasoning results in two additional conjectures:

2. Larger values of *k* lead to a less accurate approximations of distances.
3. In general, shorter distances will be better preserved than longer distances.

**Fig. 2:**
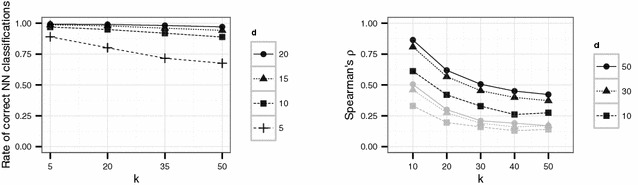
Influence of Lipschitz embedding on two different data analysis tasks. The *left* plot shows the rate of correct nearest neighbour classifications depending on parameters *d* and *k* of the Lipschitz embedding. The *right* plot shows the Spearman correlation $\rho$ between original and approximated distances for different choices of *d* and *k* of all distances in the data files to two fixed points. The *black lines* refer to a fixed point in the centre of England whereas the *grey lines* refer to a fixed point in the north of England

**Fig. 3:**
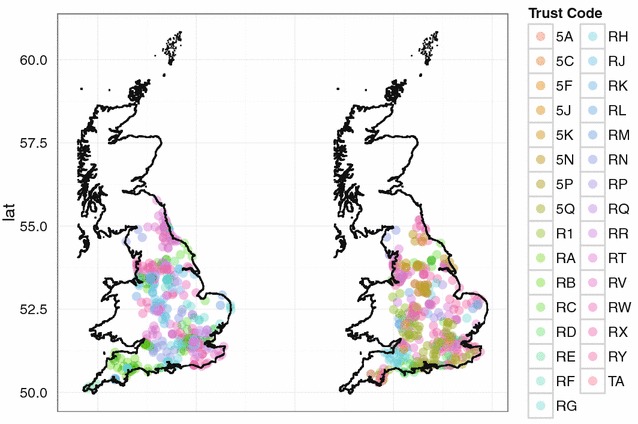
Target (*left*) and identification file (*right*) in the first scenario (English hospital data). Each data file consists of 400 geocoded hospitals and the truncated trust code as quasi-identifier. Different colours refer to different trust codes

These effects will be demonstrated by an example of three pairs of British cities with different spatial distances: Liverpool–Manchester ($50 \text { km}$), London–Sheffield ($228 \text { km}$) and Plymouth–Newcastle ($540 \text { km}$). Figures 1, 2, 3 in Additional file 1: Appendix show the conjectured effects for these distances using embedding parameters $k \in \{1,3,5\}$ and $d \in \{20, 100, 500\}$. Each combination of parameters was replicated in 100 embeddings. The plots show kernel density estimators of the approximated distances. The plots support both conjectures: Increasing values of *d* decrease the deviation of approximated distances; therefore the approximations are closer to the original distances. The same effect can be observed as *k* decreases. The third conjecture (smaller distances are much better preserved than larger distances) is also obvious in the plots.

In general, Lipschitz embedding will result in randomly contracted distances (as already stated in Proposition 1 above). Increasing values of *k* and decreasing values of *d* will increase the variance of approximated distances. Choosing these parameters accordingly will make the recovery of the original distances for an adversary more difficult.

#### Example: influence of Lipschitz embedding on data mining tasks

In this section we consider the effect of distance modification via Lipschitz embedding on two specific data mining tasks.

For the first demonstration, we empirically determine the rate of correct nearest neighbour classifications depending on the embedding parameters *d* and *k*. Nearest neighbour classifications are essential for agglomerative cluster analysis, therefore this computation is of interest. Note that given aggregated data instead of a modified distance matrix, this computation would be impossible.

We investigated the accuracy of our method by means of a distance matrix obtained by calculating the pairwise distances between 400 randomly chosen hospitals in England. The distances between the hospitals were modified using the proposed Lipschitz embedding technique. Based on the modified distance matrix for each record its nearest neighbour was determined. We considered parameter settings with $k \in \{5,20,35,50\}$ and $d \in \{5,10,15,20\}$. For each combination of parameters 10 iterations were conducted and the average proportion of correct nearest neighbour classifications was calculated. The results of this experiment are shown in Fig. 2. Obviously, even for small values of *d* and large values of *k* (implying heavily modified distances) the proportion of correct nearest neighbour classifications is large. Only for the smallest dimension considered ($d=5$) a rapid decrease in correct classifications depending on *k* can be observed.

As second demonstration, we describe the preservation of relative orderings through the proposed variant of Lipschitz embedding. For this purpose two fixed points were chosen. One point was located in the centre and the other point at the border of the chosen geographical area. The Spearman rank correlation $\rho$ was computed between the resulting ranks for the original and the approximated distances of all points to the two selected points. Figure 2 shows the decrease of $\rho$ if *d* decreases and *k* increases. This result is in accordance with Conjectures 1 and 2 above. Conjecture 3 is illustrated by the difference between the central point and the border point. For a point at the boundary, larger distances to other points are not as accurately preserved by the Lipschitz embedding as smaller distances.

Finally, it should be mentioned that for some other methods of distance modification (for example, Kerschbaum’s method) data mining tasks such as this cannot be computed at all.

### Empirical privacy analysis of distance matrices

Standard methods for the evaluation of privacy of distance matrices seem to be unavailable.^2^ Therefore, we use a recently published de-anonymisation attack based on graph matching [29].

#### A graph theoretic linkage attack

The graph theoretic linkage attack described here is related to a general attack mode termed *linkage attack* which is widely discussed in the literature on statistical disclosure control [3]. Linkage attacks assume access to an identified auxiliary microdata file by the attacker. By comparing common attributes of this auxiliary file (termed the *identification file*) with the published microdata file (termed the *target file*), the attacker tries to match the records of both files. In this context, common attributes in both files (such as sex, age, ethnicity in the case of personal microdata) are referred to as *quasi-identifiers* [30] or *indirect identifiers* [9].

The power of linkage attacks using quasi-identifiers has been demonstrated repeatedly (for an example, see [31]). The most popular demonstration is due to Sweeney [32]: She was able to detect the record corresponding to the governor of Massachusetts in a published health data file by linking it with a publicly available voter registration list. Recently, some theoretical results on linkage attacks have been derived [33].

To prevent linkage attacks, many of the well-known anonymisation techniques for microdata modify the original data. For instance, the R package sdcMicro [34] provides such strategies for the anonymisation of tabular data. Probably the most popular strategy used to prevent unambiguous linkage is provided by the concept of *k*-anonymity [32]. A microdata table satisfies *k*-anonymity if each record cannot be distinguished from at least $k-1$ other records by means of the quasi-identifiers. Therefore, a microdata file is *k*-anonymous if in case that an entity from the identification file can be linked with a given record from the target file, then it can be linked with at least $k-1$ other records as well.

Generally, the set of all potential matches between target and identification file (which we denote with *V* hereafter) contains correct matches (true positives) as well as incorrect ones (false positives). When an entity *i* from the identification file can be matched with more than one observation unit *t* of the target file, different potential matches are equally likely.

However, when additional information about the distances between the units of observation in the target file is released and the attacker can calculate the distances between the entities in the identification file, the compatibility of matches in *V* can be examined by the attacker. By definition, an element of *V* is a pair (*t*, *i*) where *t* is a unit of observation from the target file and *i* a known entity from the identification file such that *t* and *i* coincide concerning the released attributes. Therefore, the pair (*t*, *i*) corresponds to a potential match.

Given two such pairs $(t_1,i_1)$ and $(t_2,i_2)$, the attacker has to decide whether these pairs are *compatible*. This decision can only be made based on the knowledge of the released distance between $t_1$ and $t_2$ (which is possibly modified or perturbed by the data holder) and the knowledge of the proper distance between $i_1$ and $i_2$.

The precise definition of compatibility is critically dependent on the way the distances between the observation units have been modified by the data holder before releasing the data. Below we will give a specific definition for our case of interest, where the distances are modified by the variant of Lipschitz embedding introduced in the “Methods” section.

The result of checking all potential matches of *V* in terms of their compatibility can be modelled by means of a simple undirected graph $G=(V,E)$. The vertex set *V* of this graph is just the set of all potential matches as above. The edge set *E* is defined as follows: two matches are tied by an edge (i.e., the corresponding vertices are adjacent) if and only if they are classified as compatible. From now on, we refer to this graph as the *compatibility graph*.^3^

Having constructed the compatibility graph, the attacker will try to find a set *C* of vertices in *V* of maximum size such that any two vertices from *C* are adjacent. In the field of algorithmic graph theory, this problem is referred to as the *maximum clique problem* [35]. The adversary will consider the matches corresponding to the vertices of such a maximum clique *C* and drop other potential matches.^4^ The maximum clique problem for a given graph is known to be NP-hard. Therefore, the development of techniques for solving this problem exactly or at least approximately [35] has received a lot of attention in the literature. For the computational experiments in the “Empirical privacy analysis of distance matrices” section, we used the C++ implementation of the exact maximum clique detection algorithm introduced by Konc and Janežič in [36].

We give a definition for the compatibility of two matches when the released distances between the units of the target file are modified by the proposed Lipschitz embedding. Consider $(t_1,i_1), (t_2,i_2) \in V$. The attacker has direct access to $\widetilde{d}(t_1,t_2)$ only and not to $d(t_1,t_2)$; knowledge of the latter would permit him to compare $d(t_1,t_2)$ and $d(i_1,i_2)$ directly: if $d(t_1,t_2) \approx d(i_1,i_2)$ the matches $(t_1,i_1)$ and $(t_2,i_2)$ would be classified as compatible and $(t_1,i_1)(t_2,i_2)$ would be taken into the edge set *E*. If only $\widetilde{d}(t_1,t_2)$ is known, the attacker can use a different strategy. In this setting the Lipschitz embedding of $i_1, i_2$ into $\mathbb R^d$ is repeated many times for the current parameter values *d* and *k* to estimate the distribution $\widetilde{d}(i_1,i_2)$.^5^ This estimated distribution of $\widetilde{d}(i_1,i_2)$ can finally be compared with the known realization of $\widetilde{d}(t_1,t_2)$. This can be seen as constructing an empirical $\alpha$-*tolerance interval* by taking the smallest (with respect to its length $u-l$) interval $[l,u]_{i_1, i_2}$ which contains at least the proportion $\alpha \in (0,1)$ of the simulated realisations of $\widetilde{d}(i_1,i_2)$. Using this approach, an attacker might define the matches $(t_1,i_1)$ and $(t_2,i_2)$ as compatible if and only if $\widetilde{d}(t_1,t_2)\in [l,u]_{i_1, i_2}$.

Note that this way of attack has large computational costs, because Monte Carlo experiments have to be performed for many pairs of points from the identification file. Therefore, this is only reasonable for moderate sizes of *V*.

#### A simulation to study the privacy preserving properties of the Lipschitz embedding

Since the contractive properties of the proposed method are well understood, now the privacy properties of the embedding have to be studied. For this, we conducted simulation studies on the basis of two different scenarios. In the first scenario, we performed a simulation study based on a target and an identification file from a dataset of 847 geocoded hospitals in England.^6^ We sampled 400 records for each file with an overlap of 40 hospitals belonging to both files. As quasi-identifier the TrustCode^7^ was selected. To make assignments of the vertex labels more difficult, only the first two characters of the trust code were used. In the second scenario, we conducted the same experiments on a target and an identification file consisting both of 500 simulated records on individuals from Germany with an overlap of 50 records. In this scenario, sex and age were chosen as quasi-identifiers.

We assume no measurement errors and no missing values in either data set. Therefore, if a sampling unit is part of the target and identification file, both the geographic coordinates and the quasi-identifiers match. This assumption of perfect background knowledge for the attack is very conservative, since better information makes re-identification more likely. However, it seems to be more appropriate to err on the conservative side and be protective, rather than permissive, with potentially sensitive data (see [4], p. 127).

**Fig. 4:**
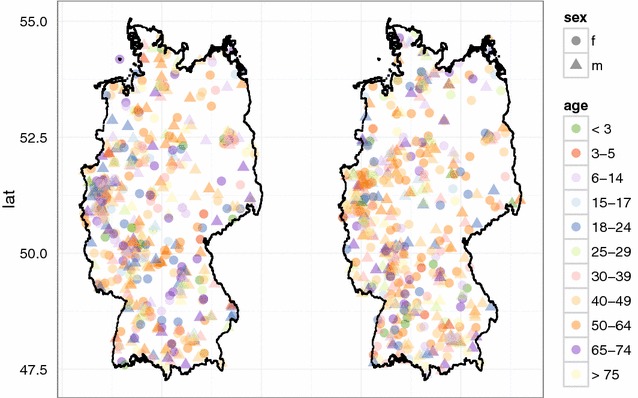
Target (*left*) and identification file (*right*) in the second scenario (simulated German population data). Each data file consists of 500 geocoded German addresses and sex and age as quasi-identifiers

A visualisation of the target and the identification files for the two scenarios considered are given in Figs. 3 and 4, respectively. Note that the vertex set *V* of the compatibility graphs contains 7976 (resp. 15517) nodes of which only 40 (resp. 50) correspond to true matches. For this reason, performing a classical linkage attack in both scenarios is not promising and the target files would certainly be regarded as sufficiently anonymised if no additional distance information had been released.

The linkage attack was repeated using the data sets described above for different values of *k* and *d*.

In a first experiment, we set the parameters $d \in \{20,100,500\}$ and $k \in \{1,\ldots ,10\}$. The threshold $\alpha$ varied between 0.1, 0.5 and 0.9. For each parameter combination, the simulation (consisting in the generation of $\widetilde{D}$, generation of the compatibility graph, maximum clique search and extraction of the corresponding matches) was repeated 20 times.

In a second experiment, we used parameter values which should yield higher levels of protection than the set of parameter in the first experiment. Therefore, we considered $d \in \{ 20, 60, 100 \}$ and $k \in \{10,12,14,\ldots ,30\}$. Here, we used $\alpha = 0.1$ and $\alpha = 0.5$ for the threshold parameter $\alpha$. Again, for each parameter setting the simulation was repeated 20 times.

Data preparation and analysis were done with the statistical programming language R [22]. As indicated above, we used the C++ implementation of the maximum clique detection algorithm proposed in [36] which is available from http://www.sicmm.org/~konc/maxclique/. The number of iterations was set to 20.000.000; the maximal clique found until this iteration was used as result.

Success of the attack was quantified with *precision* ($\text {prec}$) and *recall* ($\text {rec}$), the most widely used measures for data linkage processes [37]. For example, [38] used precision and recall as measures in an evaluation of automatic de-identification procedures. Further studies of this type using the same measure are reported by [39].

Here, $\mathbf {TP}$ denotes the number of successful re-identifications, $\mathbf {FP}$ the number of false assignments and $\mathbf {FN}$ the number of common entities of target and identification file that were not detected by the attack. Precision and recall are defined by

$$\begin{aligned} \text {prec}=\frac{\mathbf {TP}}{{\mathbf {TP}}+{\mathbf {FP}}} \quad \text { and } \quad \text {rec}=\frac{{\mathbf {TP}}}{{\mathbf {TP}}+{\mathbf {FN}}}. \end{aligned}$$

Note that in our framework the attacker would primarily be interested in attaining high precision, implying a high proportion of true positives among all assignments. This is due to the fact that correct re-identification of some entities would permit the re-identification of additional entities. Therefore, we focus on precision to measure the attacker’s success. Accordingly, $1-\text {prec}$ can be interpreted as a measure of the empirically attained anonymity. However, we will also report briefly results concerning recall as a measure of which proportion of the overlap of both files can be detected by the attacker.

## Results

**Fig. 5:**
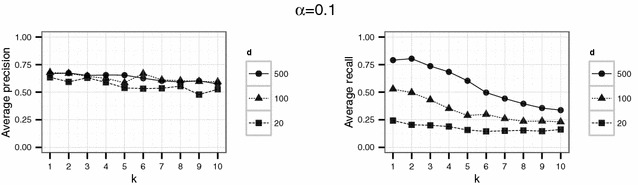
Results of the first experiment for $\alpha =0.1$ for the English hospital data

**Fig. 6:**
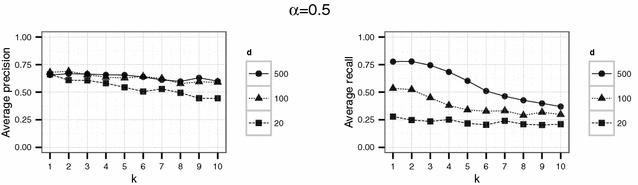
Results of the first experiment for $\alpha =0.5$ for the English hospital data

**Fig. 7:**
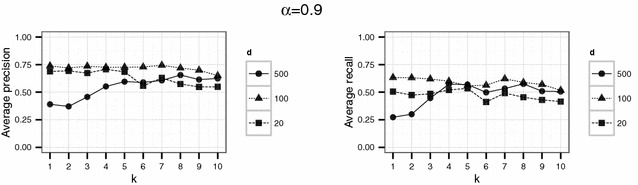
Results of the first experiment for $\alpha =0.9$ for the English hospital data

Using the parameter settings of the first experiment for the English hospital data, Figures 5, 6, 7 show high levels of success for the attack for all parameters considered. Hereby, we demonstrated the practical utility of the proposed attack: For unsuitable parameter settings as used here, the attack will yield successful re-identifications. The attacker can achieve a precision of nearly 0.5 (50 % of her re-identifications are correct if she chooses $\alpha =0.5$) given even the most secure parameter settings considered in the first experiment ($k=10$ and $d=20$) (Fig. 6). This level of precision will be unacceptable for sensitive data in most applications.

However, a decrease in precision with increasing *k* is obvious for all but the largest number of dimension *d* considered here. For the smallest number of dimensions ($d=20$), precision decreases rapidly with increasing *k*.

**Fig. 8:**
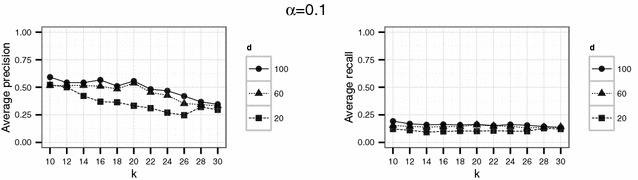
Results of the second experiment for $\alpha =0.1$ for the English hospital data

**Fig. 9:**
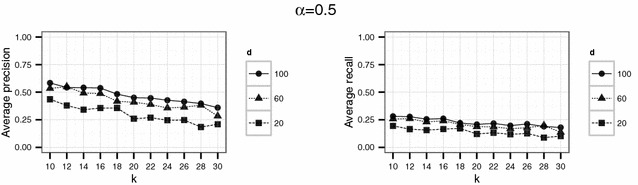
Results of the second experiment for $\alpha =0.5$ for the English hospital data

To investigate if higher levels of anonymity can be achieved by the proposed Lipschitz embedding, higher values of *k* ($k \in \{10,12,\ldots ,30\}$) were used in the second experiment. For the dimension parameter $d \in \{20, 60, 100\}$ was chosen. The results for this settings are shown in Figs. 8 and 9.

For all levels of *d* considered, the precision approaches $25~\%$ for large *k*. Additional tests using even larger values of $k \in \{50,100\}$ did not result in a further decrease of precision.

**Fig. 10:**
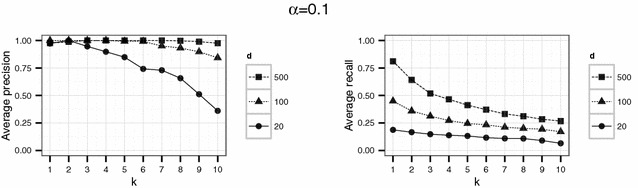
Results of the first experiment for $\alpha =0.1$ for the German population data

**Fig. 11:**
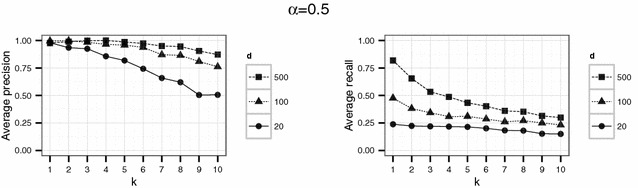
Results of the first experiment for $\alpha =0.5$ for the German population data

**Fig. 12:**
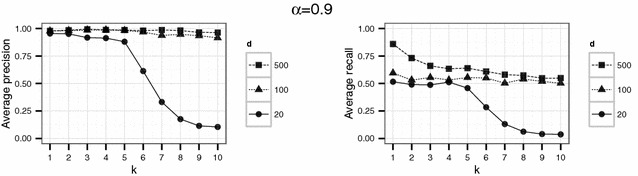
Results of the first experiment for $\alpha =0.9$ for the German population data

**Fig. 13:**
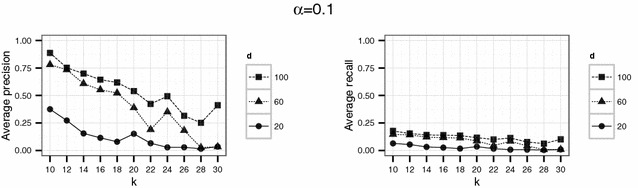
Results of the second experiment for $\alpha =0.1$ for the German population data

**Fig. 14:**
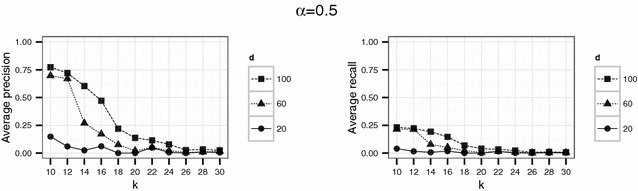
Results of the second experiment for $\alpha =0.5$ for the German population data

This may be due to the limited size of the compatibility graph. This reasoning is based on the results obtained with the slightly larger files for the German population data in the second scenario. On the German data, the embedding method results in a failure of the graph theoretic linkage attack: Whereas the attack achieves a certain amount of successful re-identifications for the first experiment (see Figs. 10, 11, 12), only a precision close to 0 can be achieved by the attack for suitable parameter choices in the second experiment (see Figs. 13 and 14). A natural explanation for this observed difference in attainable precision between the two scenarios is the difference in size of the compatibility graphs (15517 for the German population data compared to 7976 nodes for the English hospital data).

For the English hospital data, the results regarding the recall are similar to the results on precision. Whereas in Figs. 5 and 6 recall decreases with *k* at least for the largest number of dimension considered ($d=500$ in this case), the recall decreases only slightly with *d* and not with *k* for the second experiment and rarely exceeds 25 % (see Figs. 8, 9). For the German data, the recall does not approach 10 % for $d=20$ and $k \ge 20$ (see Figs. 13, 14).

However, as mentioned before, in our context recall is less important than precision since a large recall is of no use for an attacker if precision is small and correctness of the re-identification can not be evaluated. This is similar to the *k*-anonymity model, where some re-identifications might be correct, but the attacker has no way to check the truth of the identifications, since even when the attacker knows that a certain person’s record belongs to the target file, an assignment of this person to a record will be correct with probability $\le 1/k$. Therefore, by replicating each simulation step 20 time, we aim to approximate the probability 1/*k* by generating an expected value for precision.

Based on this interpretation, we consider re-identification risks of more than $25~\%$ as not suitable since this corresponds to *k*-anonymity with $k<4$. This level of protection is higher than those accepted by some European data protection agencies in practice. For example, the implementation of statistical disclosure control for the German Census [40] aims for *k*-anonymity with $k=3$. Therefore, a precision of 25 % seems to be not unreasonable for practical applications. The embedding method proposed here seems to meet this requirement.

## Conclusion

In this paper, we have introduced a new method for the modification of spatial distance matrices that protects against re-identification. The proposed method combines two desirable properties for data protection methods (see page 123 in [41]):

1. For the intended class of applications, it allows accurate statistical analyses.
2. It seems to possess the potential for high-level protection even if an adversary has in-depth background knowledge.

Due to the contractivity of the Lipschitz embedding, small distances will be preserved better than large distances. Therefore, statistical models using local features will give accurate results despite the (intended) distortion of the distance matrix. For example, agglomerative cluster analysis will give very similar results. In general, treating the transformed distance matrix as censored data might give additional options for analysis. For a comprehensive review of suitable statistical approaches for the analysis of censored data, see [42].

Of course, descriptive statistics based on the modified distance matrix alone will be biased. However, with the exception of quantiles, publishing additional descriptive statistics of the unmodified distance matrix seems not to increase the re-identification risk for the graph based attack since this information is not used in the attack. Since no other attack on distance matrices is known at the moment, there is no way to assess the risk for unknown attack methods.

For one of our example data sets, we have shown that the only currently known attack on distance matrices fails if the embedding parameters are chosen carefully. For this data set consisting of simulated German population data, choosing $d=20$ and $k=30$ resulted in very few successful re-identifications. Of course, for a given data set, the data custodian has to determine appropriate values for *d* and *k* by simulations. However, similar considerations are necessary for all other geo-masking methods.

We consider the levels of privacy protection reported here as conservative estimates, since real world attacks will suffer from practical obstacles such as measurement and data processing errors in distances. Furthermore, the amount of overlap between target and identification file will often be lower, resulting in lower precision and recall of an attack. Finally, for large files, the graph theoretical linkage attack becomes computationally expensive since the attack requires exponential resources with increasing size of the compatibility graph.

However, the privacy analysis presented here is based only on the graph theoretic linkage attack. To our knowledge, no other attacks on distance matrices have been published. Therefore, for a detailed evaluation of disclosure risks for publishing distance matrices further research on attacks is needed.

## Additional file

10.1186/s12942-015-0031-7 This file contains an exemplary implementation of the proposed method in the statistical software R. Moreover, it illustrates the effect of the proposed method on the preservation of distances using the example of three pairs of English cities corresponding to a small, a moderate and a large distance, respectively.

## Footnotes

1 In [43], a method using Monte Carlo simulations is proposed for this problem. Nevertheless, the distance approximations resulting from this method will impede clustering procedures.

2 Attacks and attacking methods for (medical) research data are rarely published [44]. Geographical information in these attacks is used as quasi-identifiers [45], not as distance information. In the context of health data, to the best of our knowledge there is only one (roughly) reported attack method using distance information [4].

3 For a more detailed account of the definition of the compatibility graph, we refer the reader to [29], where an example for constructing a compatibility graph (termed *product graph* in that paper) is given.

4 If the maximum clique is not uniquely determined we assume that the adversary chooses one maximum clique at random.

5 We make the conservative assumption that the parameters are known to the attacker. If the parameters are unknown, re-identification becomes even harder, because the parameters *d* and *k* have to be estimated.

6 This dataset is available at http://www.whatdotheyknow.com/request/list_of_hospitals.

7 The National Health Service Act 2006 created NHS trusts “(...) to provide goods and services for the purposes of the health service”. The codes are available at http://data.gov.uk.
