# Supplementary material for: Anonymisation of geographical distance matrices via Lipschitz embedding
Source: Int J Health Geogr. 2016 Jan 7;15:1. doi: 10.1186/s12942-015-0031-7 (PMC4704375; doi:10.1186/s12942-015-0031-7)
Supplement: Supplementary file 1 — 10.1186/s12942-015-0031-7 This file contains an exemplary implementation of the proposed method in the statistical software R. Moreover, it illustrates the effect of the proposed method on the preservation of distances using the example of three pairs of English cities corresponding to a small, a moderate and a large distance, respectively. [file 12942_2015_31_MOESM1_ESM.pdf]

Web appendix for the article

Anonymisation of geographical distance matrices via Lipschitz embedding

in the *International Journal of Health Geographics*

Martin Kroll and Rainer Schnell
December 18, 2015

1 Implementation of the proposed method

In this section we give an example workflow using R that demonstrates the use of the proposed method. In this demonstration, an approximate version of the distance matrix between the 10 largest English cities is created. The elements of the reference sets are sampled from the area of the United Kingdom.

1.1 Loading packages and auxiliary data

We begin by loading the necessary package `sp` and a SpatialPolygon object that represents the boundary of Great Britain.

```
> library(sp)
> load("GBR_adm0.RData") # loads gadm
> # this file can be downloaded from
> # http://biogeo.ucdavis.edu/data/gadm2/R/DEU_adm0.RData
> # many other Global Admininstrative Areas available there
>
> crs.string<-proj4string(gadm)
> print(crs.string)

[1] " +proj=longlat +ellps=WGS84 +datum=WGS84 +no_defs +towgs84=0,0,0"
```

1.2 Loading the demonstration data

Our demonstration data is a data.frame containing the ten largest cities in England and their corresponding geographic coordinates with respect to the reference system WGS 84.

```
> load("cities.RData")
> head(cities)

  city           lon        lat
1     London, UK -0.1277583 51.50735
```

```
2  Birminham, UK -1.8904010 52.48624
3      Leeds, UK -1.5490774 53.80076
4  Sheffield, UK -1.4700850 53.38113
5   Bradford, UK -1.7593980 53.79598
6 Manchester, UK -2.2426305 53.48076
```

1.3 Transform WGS 84 coordinates into a SpatialPoints object

To use the methods provided by the R package `sp`, the object cities (of type data.frame) have to be transformed into an object of type SpatialPoints. Because we want to calculate distances between these points and the reference points which will later be sampled from `gadm`, we have to choose the same geographic projection (which is given by `crs.string`).

```
> sp.cities<-SpatialPoints(cities[,2:3],proj4string=CRS(crs.string))
> head(sp.cities)

SpatialPoints:
     lon        lat
[1,] -0.1277583 51.50735
[2,] -1.8904010 52.48624
[3,] -1.5490774 53.80076
[4,] -1.4700850 53.38113
[5,] -1.7593980 53.79598
[6,] -2.2426305 53.48076
Coordinate Reference System (CRS) arguments: +proj=longlat
+ellps=WGS84 +datum=WGS84 +no_defs +towgs84=0,0,0
```

1.4 Definition of embedding parameters

We choose the embedding parameters d and k.

```
> d<-20 # dimension parameter
> k<-5 # size of reference sets
```

1.5 Lipschitz embedding

Now, the Lipschitz embedding can be performed. Hereby each of the ten cities is mapped into $\mathbb{R}^{20}$.

```
> N<-length(sp.cities)
> lipschitz.coordinates<-matrix(0,nrow=N,ncol=d)
>
> for(i in 1:d){
+  reference.set<-spsample(gadm,k,type="random",iter=+Inf)
+  temp<-spDists(sp.cities,reference.set,longlat=TRUE)
+  temp<-apply(temp,1,min)
+  lipschitz.coordinates[,i]<-temp
+ }
>
> print(lipschitz.coordinates[1,])

[1]   91.72952 270.43442  20.23935  67.10500  30.60395 261.92201 123.64158
[8]   41.36210  50.98461  99.27907  49.17343  37.20873  19.98669  29.99003
[15] 110.35535 124.32715  66.49502  70.41790 212.67272 183.10468
```

1.6 Compute approximate distances

Finally, the distance matrix between the embedded cities remains to be calculated.

```
> D.approx<-dist(lipschitz.coordinates,
+   method ="maximum",diag=TRUE,upper=TRUE)
> D.approx<-as.matrix(D.approx)
```

2 Effect of Lipschitz embedding on the distance between three pairs of cities

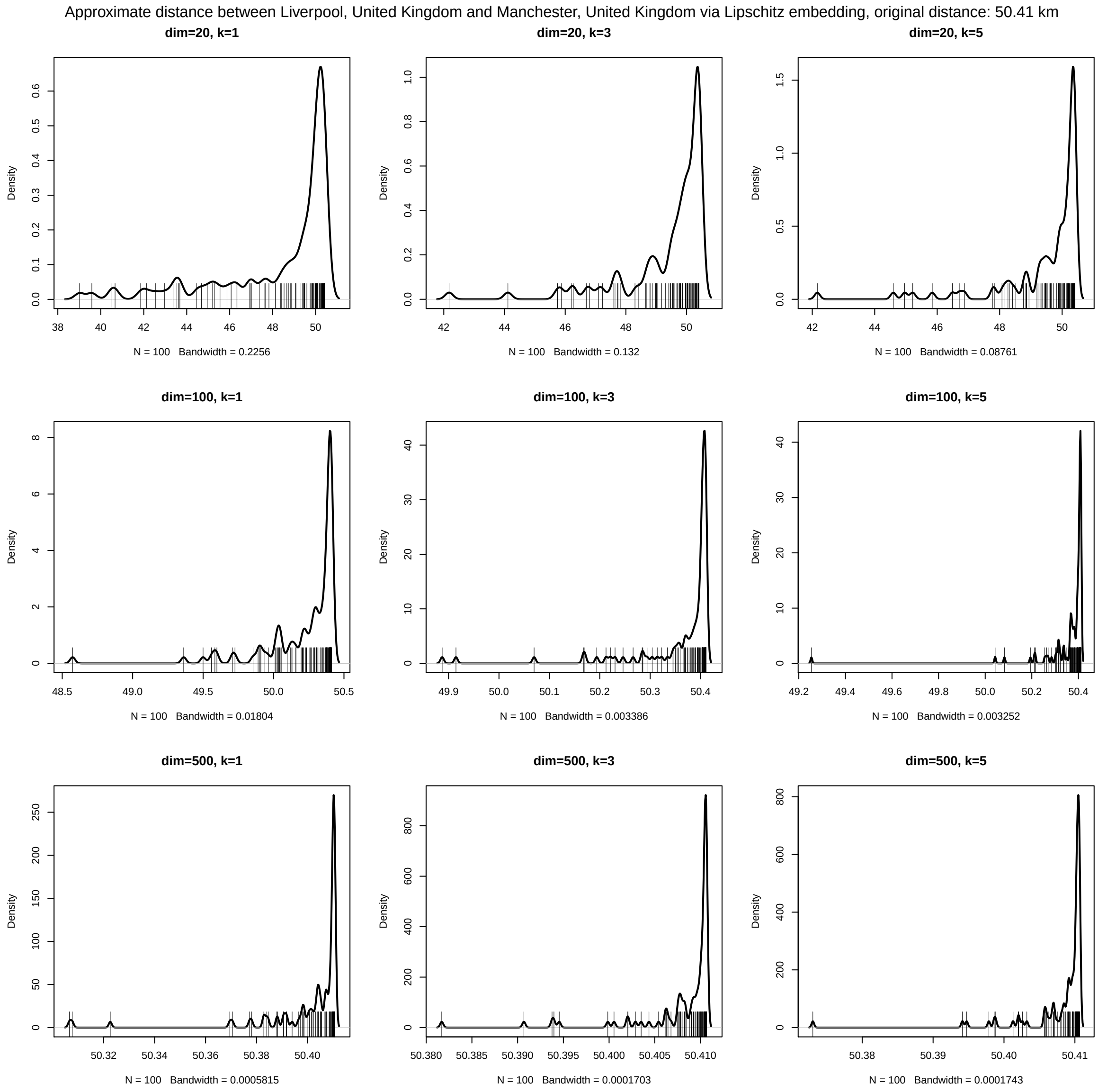

Figure 1: Approximations of a short distance (50 km, Liverpool – Manchester) for different parameter choices.

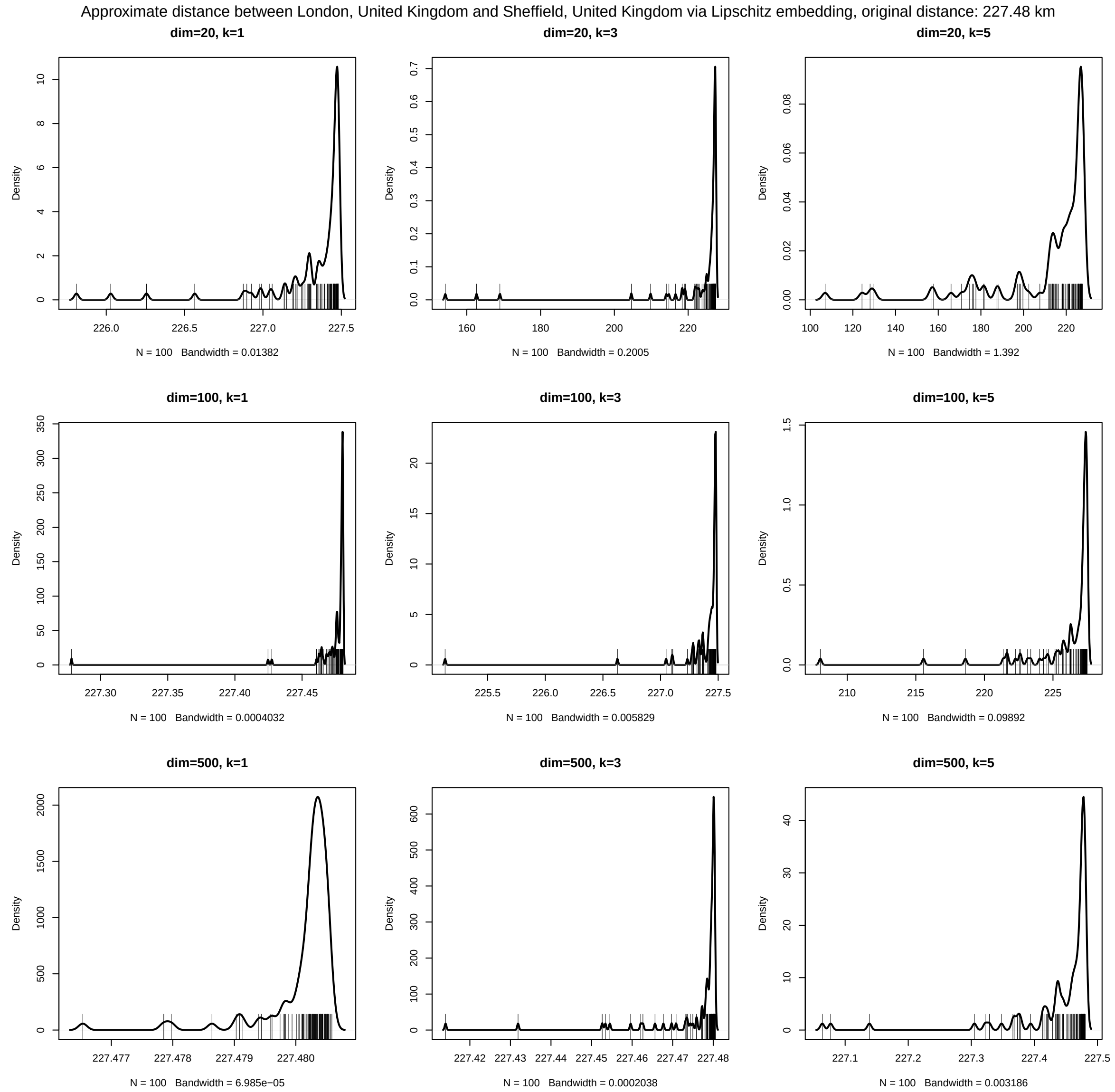

Figure 2: Approximations of a medium distance (228 km, London – Sheffield) for different parameter choices.

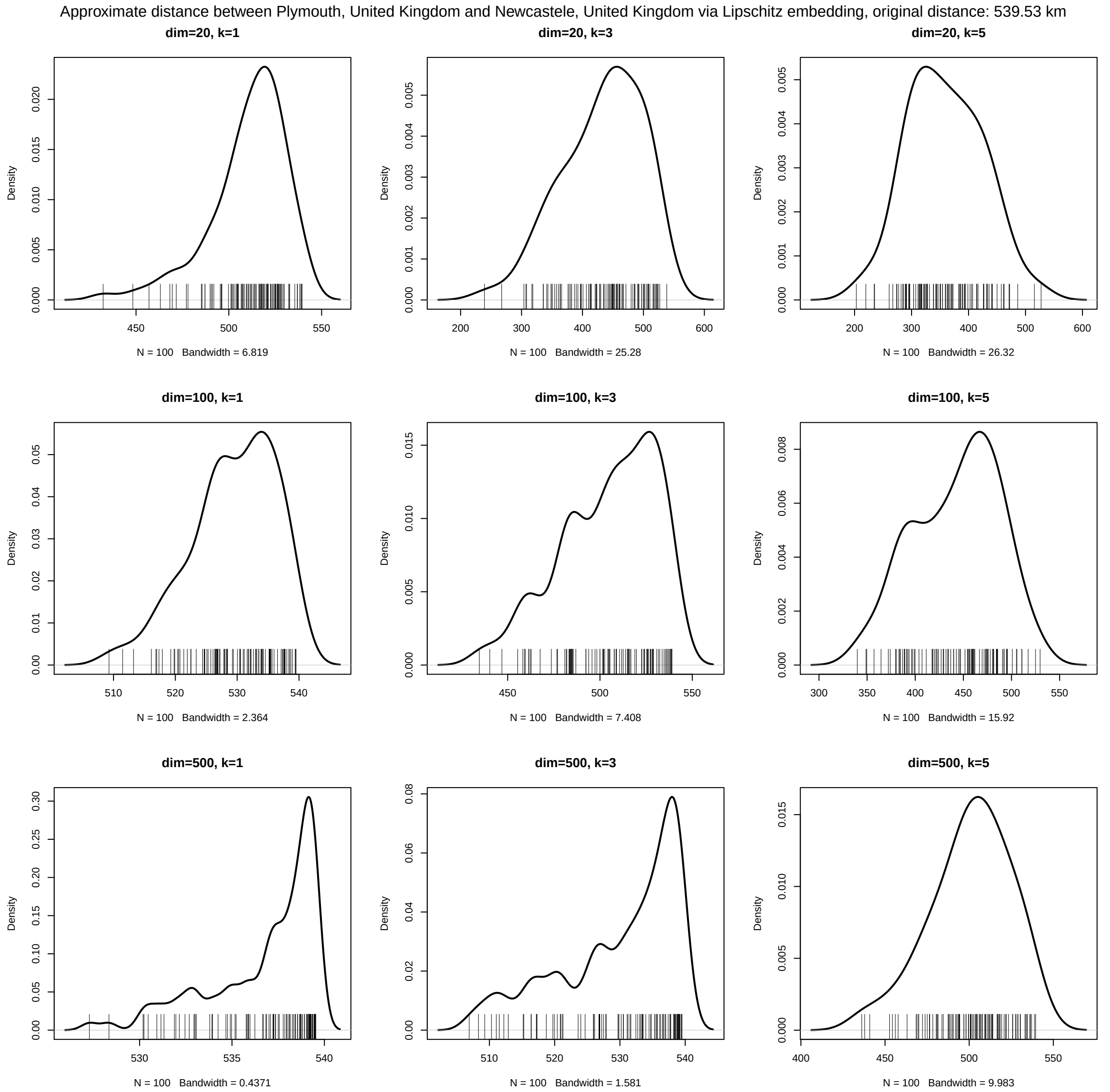

Figure 3: Approximations of a long distance (540 km, Plymouth – Newcastle) for different parameters choices.
